# A Scale-Invariant Looming Detector for UAV Return Missions in Power Line Scenarios

**DOI:** 10.3390/biomimetics10020099

**Published:** 2025-02-10

**Authors:** Jiannan Zhao, Qidong Zhao, Chenggen Wu, Zhiteng Li, Feng Shuang

**Affiliations:** 1 Guangxi Key Laboratory of Intelligent Control and Maintenance of Power Equipment, School of Electrical Engineering, Guangxi University, Nanning 530004, China; jzhao@gxu.edu.cn (J.Z.); 2212301082@st.gxu.edu.cn (Q.Z.); 2212401020@st.gxu.edu.cn (Z.L.); 2 State Grid Lishui Power Supply Company, Grid Zhejiang Electric Power Company, State Grid Corporation of China, Lishui 323000, China; 213143554@seu.edu.cn

**Keywords:** bioinspiration, looming-object sensitive neural network, small-target attention, UAV power line detection

## Abstract

Unmanned aerial vehicles (UAVs) offer an efficient solution for power grid maintenance, but collision avoidance during return flights is challenged by crossing power lines, especially for small drones with limited computational resources. Conventional visual systems struggle to detect thin, intricate power lines, which are often overlooked or misinterpreted. While deep learning methods have improved static power line detection in images, they still struggle with dynamic scenarios where collision risks are not detected in real time. Inspired by the hypothesis that the Lobula Giant Movement Detector (LGMD) distinguishes sparse and incoherent motion in the background by detecting continuous and clustered motion contours of the looming object, we propose a Scale-Invariant Looming Detector (SILD). SILD detects motion by preprocessing video frames, enhances motion regions using attention masks, and simulates biological arousal to recognize looming threats while suppressing noise. It also predicts impending collisions during high-speed flight and overcomes the limitations of motion vision to ensure consistent sensitivity to looming objects at different scales. We compare SILD with existing static power line detection techniques, including the Hough transform and D-LinkNet with a dilated convolution-based encoder–decoder architecture. Our results show that SILD strikes an effective balance between detection accuracy and real-time processing efficiency. It is well suited for UAV-based power line detection, where high precision and low-latency performance are essential. Furthermore, we evaluated the performance of the model under various conditions and successfully deployed it on a UAV-embedded board for collision avoidance testing at power lines. This approach provides a novel perspective for UAV obstacle avoidance in power line scenarios.

## 1. Introduction

The evolution of the smart grid necessitates advanced detection technologies, including climbing robots, helicopters, and unmanned aerial vehicles (UAVs). Among these, UAVs are the most widely employed platform due to their low cost, high efficiency, and precise detection capabilities [1]. However, current UAVs normally rely on pre-determined flight routes [2,3], while in emergency cases or customized commanded return missions, they still lack the adaptability to address dynamic collision avoidance, especially towards crossover power lines.

Power grid companies have reported frequent crashes of detection drones into power lines during return missions [4]. This issue arises from the difficulty in real-time sensing of the collision risk from thin power lines that lack distinct texture features [5]. Existing approaches to UAV collision detection, such as energy-intensive laser scanning [6], traditional methods relying on detectable object textures [7], or deep learning-based power line detection models [8,9,10], still face significant limitations. These limitations are compounded by the complexity of network architectures, heavy reliance on large datasets, and hardware constraints related to power management and payload capacity [11,12]. These methods cannot be used in direct application for fast collision warning for high-speed motion in power line scenarios.

In nature, insects demonstrate the potential of motion vision for perceiving and navigating complex environments. Despite their compact visual systems, insects can efficiently forage, avoid predators, and locate mates in dynamic settings [13,14,15]. This remarkable perceptual efficiency has spurred significant research interest in motion vision. Inspired by insect vision, computationally efficient solutions—such as optical flow [16], event cameras [17], and looming-object detection (i.e., identifying objects on a collision course)—have been proposed [18]. Notably, bio-inspired looming-object detection has emerged as a promising collision warning approach in dynamic, complex environments, with demonstrated applications in robotic systems. In mobile robotics, ref. [19] investigated the application of collision avoidance and orientation selection capabilities of biological neurons in sensor designs, demonstrating successful obstacle avoidance behavior through experiments with Lego block towers. To address challenges in complex backgrounds and varying illumination, ref. [20] proposed the grouping layer structure combined with a feature fusion module, enabling small robots to avoid multi-shaped obstacles such as wooden blocks and inverted-V structures. For algorithmic optimization, ref. [21] employed genetic algorithms to refine system parameters, validating their approach in automotive scenarios and inflatable car collision experiments, thereby establishing a novel framework for reliability assessment. In UAV obstacle avoidance, ref. [22] integrated two Lobula Giant Movement Detector (LGMD) models to achieve mutual collision avoidance in both bright and dark environments. Building on this, ref. [23] synergized moment-based LGMD with deep reinforcement learning, empowering drones to stably avoid cabinets, rocks, and trees while minimizing visual jitter in simulated scenes. For dynamic environments, ref. [24] introduced a distributed presynaptic connection (DPC) mechanism to improve looming-object selectivity in complex scenes, though its detection capability remains limited for small-scale obstacles. Conversely, ref. [25] developed a dedicated line-attention module to detect dynamic power lines in real-flight first-person view videos, but this method exhibits excessive looming-object sensitivity to linear features. These studies neglect research on multi-scale looming objects, including small-scale obstacles (e.g., power lines [26]).

**Figure 1:**
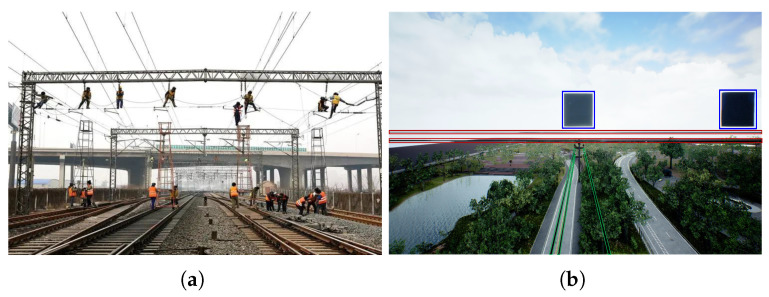
(**a**) Example of a complex power line detection scenario in the real world. (**b**) A simplified abstraction of the complex power line detection scenario for unmanned aerial vehicles (UAVs). Key elements are split into four categories: The tracking lines (green), obstacles (blue), the horizontal lines (red), and the background (unlabeled).

In looming-object detection, Lobula Giant Movement Detector (LGMD) [27] is not particularly sensitive to object size, but its detection of looming-object contours relies on the assumption that the contours of looming targets have a high degree of aggregation. This assumption is based on the idea that the edge contours of regular objects appear coherent and thick in motion vision due to higher motion speeds when approaching, whereas background motion typically produces sparse and incoherent visual stimuli [20]. In a previous study, we enhanced the model by incorporating a linear high-speed contour enhancement technique, referred to as looming powerline detection (LPD) [25]. This method allows for the selective extraction of features generated by linear targets from sparse contours. However, this attentional mechanism [28] also inadvertently filters out nonlinear objects, limiting its ability to simultaneously detect obstacles at multiple scales, which is inadequate for application. In a return mission, the UAV may face cross over power lines, trees, buildings, and even maintenance workers on other lines (e.g., Figure 1a). Therefore, the collision risk from all these normal and abnormal-sized obstacles needs to be noted. For the image processing model, it is difficult to acquire cross-size attention ability. If we abstract the elements of this complex task, an analogous scenario is depicted in Figure 1b. In this scenario, the LPD can detect looming power lines (highlighted in red) but is insensitive to the central threatening cube. LGMD with the introduction of the distributed presynaptic connection mechanism (D-LGMD) [24], on the other hand, can only respond to the two normal-sized cubes (highlighted in blue), with a stronger response to the non-threatening cube on the right compared to the central threatening cube.

Additionally, negative factors such as camera ego-motion and location-induced sensitivity variations have not been addressed. In the literature, extensive research has focused on enhancing bio-inspired looming-object detection algorithms, particularly in mitigating ego-motion-induced noise. One common approach is to estimate and compensate for camera motion using optimization techniques [29,30], though this often requires substantial computational resources. Zhao proposed the D-LGMD [24], which addresses ego-motion noise during agile UAV flights through angular velocity discrimination.

**Figure 2:**
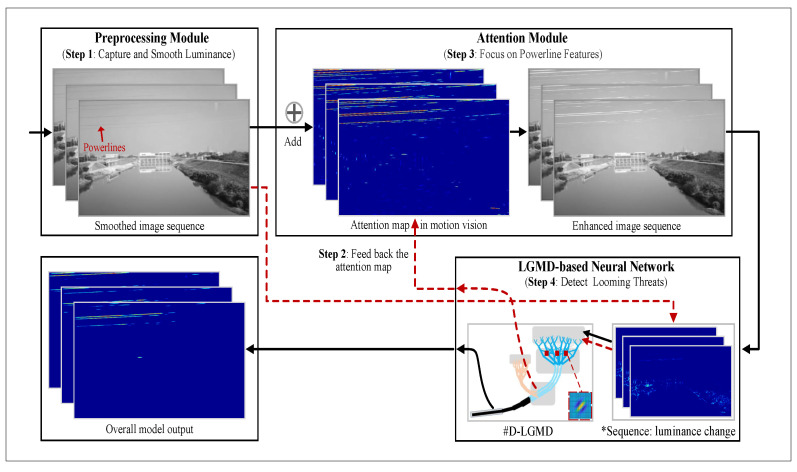
Flow diagram of the proposed visual system sensible to normal-size objects and small targets. It mainly consists of three modules: a preprocessing module (**top left**), an attention module (**top right**), and a Lobula Giant Movement Detector (LGMD)-based module (**bottom right**). There are two signal processing loops; the solid black line represents the workflow of the main processing loop, which extracts looming threat information via image velocity. The dotted red line denotes the attention loop to acquire the preferred attention to power lines. At the start, the feedback loop is processed before the looming detection loop. Note that the power lines are at the left top of images within the preprocessing module, and the small red cubes in the LGMD with the introduction of the distributed presynaptic connection mechanism (D-LGMD) module denote the kernel designed for looming power lines, which was proposed in our previous work [25]. * Sequence: Capturing image motion by recording changes in luminance within the field of view.

To address these limitations, we propose a size-invariant looming detector (SILD) with feedback attention, which expands the size-sensitivity range of the D-LGMD, enabling consistent responses to looming objects regardless of their size. The feasibility of the principle is also demonstrated in the papers [31,32]. As illustrated in Figure 2, the additive attention module enhances sensitivity to small objects (e.g., power lines) while maintaining responsiveness to normal-sized objects. Additionally, the feedback mechanism improves the precision of the stimulus response, particularly for power lines [33].

In comparison to our previous work (LPD [25]), this article achieves the following advancements:

- We introduce an additive attention feedback mechanism to enable SILD with size-invariant sensibility in looming detection, making it more practical in real-world applications.
- In terms of power line detection, the proposed SILD is advanced in efficiency and robustness when applied horizontally compared to computer vision-based and deep learning-based solutions such as the Hough transform method and D-LinkNet [8].
- Our experiments demonstrate that the proposed model can simultaneously detect thin power lines and other looming objects during real-world flights, proving its suitability for UAV looming detection in power line scenarios.
- We evaluate the model’s performance under varying backgrounds, noise conditions, and low-texture features on the Unreal Engine platform by AirSim [34], and successfully deploy it on a UAV-embedded platform to perform collision avoidance in a power line scenario.

## 2. Materials and Methods

This section elaborates on the architecture of the proposed model, which can be roughly divided into three modules: a preprocessing module, an attention module, and an LGMD-based module. As shown in Figure 2, the preprocessing module captures and smooths the input image. In contrast, the attention module enhances the weight of potential areas where line features are located using an attention map generated within the LGMD-based looming-object detector. The attention map, generated using a specialized kernel for line features denoted by small red cubes, is fed back into the attention module. The attention mechanism is implemented in an additive form so that the enhanced image retains the details of normal-sized objects but strengthens potential power lines. The enhanced image is then fed into the LGMD-based module, which discriminates the image velocity, extracting looming power lines and obstacles against background noise. It is worth noting that this section explains the mathematical framework of the proposed model, using convolution equations and more complex subtraction formulas to better simulate neurobiological signal integration. This approach connects biologically inspired modeling with practical implementation constraints. Given our goal to create a model that is both functionally computational and biologically plausible, the signaling process is best represented as a continuous integral, as factors such as the range and time delay of neurotransmitters are not discretized like the input image. In the following subsections, we first explain and analyze the location-induced uneven sensibility in Section 2.1. Then, we describe the overall neural network structure in Section 2.2 and detail its components in Section 2.3, Section 2.4 and Section 2.5.

To facilitate the reproduction of this work, the step-by-step implementation process of the proposed framework is as follows: firstly, data preprocessing is performed, i.e., the image data are read from the video frames and the Gaussian blurring technique is applied to reduce the noise. Then, the frame differences between consecutive frames are calculated to represent the motion information, followed by matrix processing for position correction of these differences. During the attention mechanism processing, horizontal and vertical line features are extracted by convolution and lateral suppression is applied and the final attention mask is used to enhance the motion regions in the original image. The DPC layer is computed to mimic the biological excitation and inhibition mechanisms of the neural layer to produce visual signals for motion detection. The grouping layer enhances the looming-object information, suppresses noise, and detects the looming threat by applying anisotropic kernels, thresholding the excitation response, and integrating to compute the membrane potential (MP). The pseudocode of the proposed framework is outlined in Algorithm 1.
**Algorithm** **1** Step-by-Step Implementation of the Proposed Framework1:**Input:** Video frames2:**Step 1: Data Preprocessing**3:    Read image data from video frames4:    Apply Gaussian blurring to reduce noise5:**Step 2: Frame Difference Calculation**6:    Compute frame differences between consecutive frames7:    Apply matrix processing for location correction8:**Step 3: Attention Mechanism Processing**9:    Extract horizontal and vertical line features using convolution10:  Apply lateral suppression to enhance motion regions11:  Generate final attention mask to enhance motion regions in the original image12:**Step 4: Distributed Presynaptic Connection (DPC) Layer Calculation**13:   Simulate biological excitation and inhibition mechanisms in the DPC layer14:   Generate visual signals for motion detection15:**Step 5: Grouping Layer Processing**16:   Apply anisotropic kernels to enhance looming information and suppress noise17:   Threshold the excitation response to filter out unwanted signals18:   Integrate the results to compute the membrane potential (MP)19:**Output:** Membrane potential (MP), indicating looming threat and triggering evasive actions

### 2.1. Analysis and Correction of Location-Induced Uneven Sensibility

This subsection explains and analyzes the location-induced bias in image motion, which diverts the looming-object detector’s response from centering on looming threats to near-miss objects. To address this issue while minimizing the computational load on the UAV’s onboard system, we propose a relatively simple solution: incorporating a correction function based on a Gaussian model (Section 2.5.1). The validity of this positional correction function is verified in Section 3.2.

**Figure 3:**
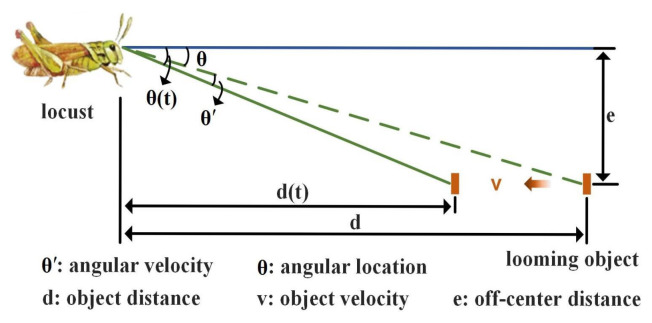
Illustration of location-induced uneven sensibility. One object with off-center distance *e* approaches the camera (locust) with forward velocity *v*. The initial state of the looming object is (θ,d), and (θ(t),d(t)) is the state of the looming object at time *t*.

In Figure 3, it can be observed from the camera’s perspective that as the looming object approaches the camera (locust), it will cross over the camera’s visual field, where the angular velocity in the visual field is determined by the object’s velocity, distance, and angular location. In other words, there is a relationship between these factors that can be described as follows:

(1) $$\begin{array}{cc} \theta (t) & =\arctan(\frac{e}{d(t)})\\ \theta^{\prime }\; & =\lim_{T_{0}\to 0}\left(\frac{\theta \left(t\right)-\theta \left(t-T_{0}\right)}{T_{0}}\right)=\frac{d\theta (t)}{dt}\\ \; & =\frac{e\cdot v}{e^{2}+d^{2}\left(t\right)} \end{array}$$

where $T_{0}$ is the camera’s sampling time interval.

As expressed in Equation (1), the angular velocity is a function of the off-center distance (e). To further clarify the relationship between them, the deviation of the angular velocity $\theta^{\prime }$ concerning the off-center distance *e* is

(2) $$\frac{\partial \theta^{\prime }}{\partial e}=\frac{(d^{2}\left(t\right)-e^{2})\cdot v}{{\left(e^{2}+d^{2}\left(t\right)\right)}^{2}}$$

It can be concluded from Equation (2) that as the off-center distance *e* increases, the angular velocity $\theta^{\prime }$ will increase in a nonlinear way when *e* is smaller than the object distance d(t) (i.e., when the angular location θ is less than or equal to 45° or when the looming object is within a 90° field of view (FOV)). Since most cameras have an FOV smaller than 90° [35], the angular velocity in the images will be a nonlinearly increasing function of the off-center distance. It is worth noting that this effect cannot be eliminated because the object distance d(t) and velocity *v* in the real world cannot be obtained from monocular camera images, and the relationship between the angular velocity and off-center distance is nonlinear, which leads to an improper higher sensitivity to near-miss movements.

### 2.2. Architecture of the Proposed Visual System

**Figure 4:**
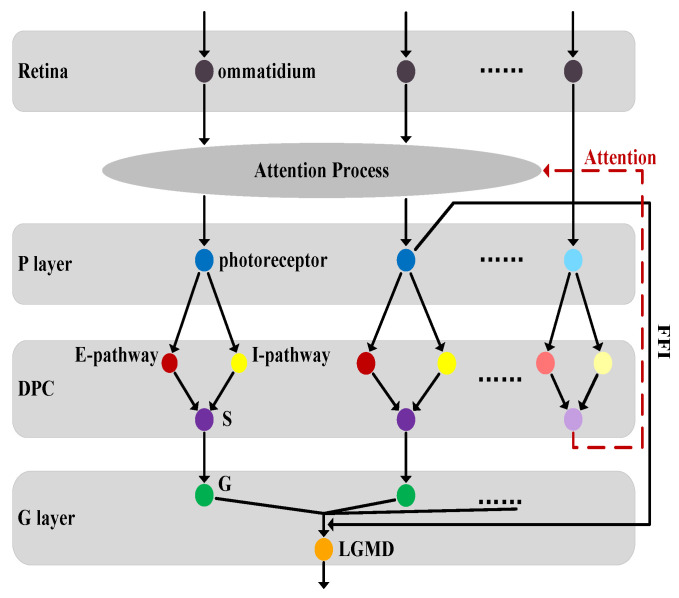
Neural network schematic of the proposed visual system. The network consists of four neural layers in sequence: retina, photoreceptor, DPC, and G layer.

Figure 4 illustrates the neural pathways in the proposed visual system, which begins with image capture by the retina and is followed by sequential processing through the photoreceptor, DPC, and G layer. The ommatidia first smooth visual information within the FOV. Then, attention is directed to regions of interest to enhance line feature signals in the image. The improved image output from the attention process contains information on power lines and normal-size objects. It is then fed into the photoreceptor layer, where visual motion is recorded and further processed by the DPC layer, including excitation and inhibition pathways. Finally, after filtering out decayed excitation, the LGMD cell groups the processed image to detect threatening obstacles, such as power lines and normal-size objects.

### 2.3. Preprocessing Module

The functionality of the preprocessing module is realized by the ommatidia in the retina layer, which captures the luminance information and smooths it using a Gaussian kernel. Formally, the preprocessing module can be defined as

(3) $$R\left(x,y,t\right)=\;\int \int \;L\left(u,v,t\right)G_{\sigma_{P}}\left(x-u,y-v\right)dudv$$

where $G_{\sigma_{P}}\left(x,y\right)$ is a Gaussian kernel with standard deviation $\sigma_{P}$:

(4) $$G_{\sigma_{P}}\left(x,y\right)=\frac{1}{2\pi {\sigma_{P}}^{2}}\exp\left(-\frac{x^{2}+y^{2}}{2{\sigma_{P}}^{2}}\right).$$

L(x,y,t) denotes the luminance of the single-channel image input at pixel (x,y) and time *t*.

### 2.4. Attention Module

**Figure 5:**
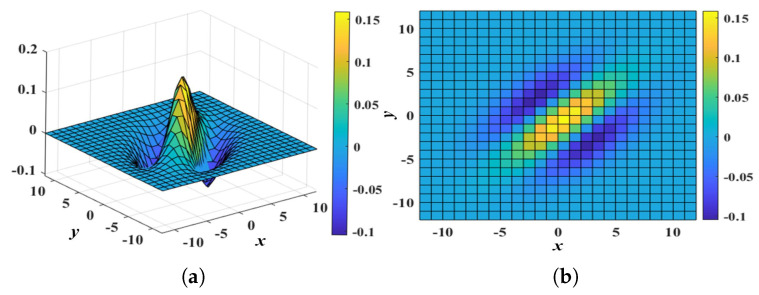
(**a**) Three-dimensional and (**b**) vertical view of an attention kernel $W_{A}\left(x,y,\sigma ,\theta ,\xi \right)$, where A=2, B=3, σ=2.0, θ= 45°, and ξ=0.5.

The attention module is utilized to improve the identification of power lines and minimize the influence of other objects, as depicted in Figure 2. This module amplifies the weight of power lines while attenuating other image elements. To achieve this, attention kernels are created according to the luminance profile of power lines. The set of attention kernels is defined using the orientation set Θ. An attention kernel is formulated as follows:

(5) $$W_{A}\left(x,y,\sigma ,\theta ,\xi \right)=A\cdot {\left[g\left(x,y,\sigma ,\theta ,\xi \right)\right]}^{+}+B\cdot {\left[g\left(x,y,\sigma ,\theta ,\xi \right)\right]}^{-}$$

(6) $$\left\{\begin{array}{c} x^{\prime }=x\;\cos\;\theta -y\;\sin\;\theta \\ y^{\prime }=x\;\sin\;\theta +y\;\cos\;\theta \\ g\left(x,y,\sigma ,\theta ,\xi \right)=\frac{\sigma^{2}-y^{\prime 2}}{\pi \sigma^{4}}\exp\left(-\frac{{\left(\xi x^{\prime }\right)}^{2}+y^{\prime 2}}{2\sigma^{2}}\right) \end{array}\right.$$

where σ, θ∈Θ, and ξ denote the size of the central part, orientation, and spatial aspect ratio, respectively. *A* and *B* are constant coefficients. ${\left[x\right]}^{+}$ and ${\left[x\right]}^{-}$ refer to max(x,0) and min(x,0), respectively. To improve the weighting of line features in images, an attention kernel is used, which can enhance the areas of interest with distinctive luminance in the slim central part compared to its bilateral sides along the orientation θ, as shown in Figure 5. The attention process involves adding the smoothed image with an attention map containing the strengthened potential line features.

(7) $$R_{s}\left(x,y,t\right)=R\left(x,y,t\right)+\eta \cdot A\left(x,y,t\right)$$

where η is the attention coefficient, and A(x,y,t) is an attention map fed back within the LGMD-based neural network, as shown in Figure 2. The luminance of regions of interest in $R_{s}\left(x,y,t\right)$ will be increased after the attention process.

### 2.5. LGMD-Based Neural Network

The exceptional performance of LGMD has drawn growing research interest, and many LGMD-derived neural networks are modeled based on related biology research. In our proposed model, the LGMD-based neural network we adopt is the D-LGMD model [24], which is competent in efficiently perceiving looming threats in the agile flights of UAVs. D-LGMD consists of three sequentially arranged neural layers, i.e., the photoreceptor layer, distributed presynaptic layer, and grouping layer. The enhanced image $R_{s}\left(x,y,t\right)$ output from the attention module is then processed in sequence by the three neural layers in the D-LGMD neural network.

The LGMD-based neural network extracts looming-object information on power lines with the attention kernel in our model. Then, the data are fed back to the additive attention module and later processed by the LGMD-based neural network to estimate looming threats, including power lines and normal-size objects. Hence, the description of the LGMD-based neural network is structurally divided into two parts: one for acquiring the attention map about power line information in the attention process and the other in the primary method for extracting looming-object information from the perceived environment information and the attention map.

#### 2.5.1. Photoreceptor Layer

The LGMD neuron is motion-sensitive and only responds to movements in the visual field. The photoreceptor layer (P layer) plays a crucial role in the LGMD neuron since it can capture image motion by recording the luminance change in the field of view. Therefore, the formulation of the P layer can be described as follows:

(8) $$P\left(x,y,t\right)=|R_{s}\left(x,y,t\right)-\int R_{s}\left(x,y,s\right)\delta \left(t-T_{0}-s\right)ds|$$

where $T_{0}$ is the sampling time interval and δ(t) is the impulse function. $R_{s}\left(x,y,t-T_{0}\right)$ and $R_{s}\left(x,y,t\right)$ are two successive images. P(x,y,t) is the output of the photoreceptor layer, revealing the luminance change from time $t-T_{0}$ to *t*.

P(x,y,t) reveals the velocity information on moving object edges. However, as shown in Section 2.1, velocity is strongly related to the looming object’s location in L(x,y,t), affecting D-LGMD’s collision risk estimation on looming objects. To address this problem, we introduce a location correction function C(x,y) (the location correction function should be a decreasing function of the distance deviated from the center (Equation (2))). Then, the rectified image motion formulation can be

(9) $$P_{C}\left(x,y,t\right)=P\left(x,y,t\right)\cdot C\left(x,y\right)$$

where C(x,y) is a deformed two-dimensional Gaussian function:

(10) $$C\left(x,y\right)=k_{1}\cdot \exp\left(-\frac{x^{2}+y^{2}}{2{\left(\frac{\epsilon /2}{n}\right)}^{2}}\right)$$

where $k_{1}$ and *n* are constants, and ϵ=max(Height,Width). Height and Width denote the number of rows and columns in L(x,y,t), respectively. ϵ is to eliminate the influence of the image size. Notably, C(x,y) is also one of the center-bias functions [36], which can help focus on the center of the image, as central information is more important than non-central information.

Accordingly, the photoreceptor layer formulation in the attention process can be expressed as

(11) $$\begin{array}{c} P_{A}\left(x,y,t\right)=|R\left(x,y,t\right)-\int R\left(x,y,s\right)\delta \left(t-T_{0}-s\right)ds|\\ P_{AC}\left(x,y,t\right)=P_{A}\left(x,y,t\right)\cdot C\left(x,y\right) \end{array}$$

where R(x,y,t) is the smoothed image in the preprocessing module at time *t*. Note that the images that the photoreceptor layer receives during the attention process are smoothed images, without being enhanced.

The photoreceptor layer allows all motion information of moving edges to be sent to the downstream neural layers without selectivity.

#### 2.5.2. DPC Layer

The distributed presynaptic connection (DPC) layer is located below the P layer and is responsible for extracting information about looming threats from all the received information from the P layer. While the P layer contains all the information on image motion, the DPC layer allows for high-speed moving edges and inhibits slow-speed ones or background noise. The DPC layer consists of three units: The excitatory pathway (E-pathway), the inhibitory pathway (I-pathway), and a linear summation unit.

In the DPC layer, the E-pathways are spatially distributed and can be expressed as

(12) $$E\left(x,y,t\right)=\;\int \int \;P_{C}\left(u,v,t\right)G_{\sigma_{E}}\left(x-u,y-v\right)dudv$$

In contrast, the I-pathways are spatially and temporally formed to abstract the pixel velocity information in the time domain and can be defined as

(13) $$I\left(x,y,t\right)=\;\int \int \int \;P_{C}\left(u,v,s\right)W_{\sigma_{I}}\left(x-u,y-v,t-s\right)dudvds$$

where $W_{\sigma_{I}}\left(x,y,t\right)$ is the spatial distribution function of the I-pathway and is described as

(14) $$W_{\sigma_{I}}\left(x,y,t\right)=G_{\sigma_{I}}\left(x,y\right)\delta \left(t-\tau \left(x,y\right)\right)$$

In Equation (14), τ(x,y) is a latency distribution function of the I-pathways in the time domain. The latency is an increasing function of the transmission distance. Then, τ(x,y) is given as

(15) $$\tau \left(x,y\right)=\alpha +\frac{1}{\beta +\exp(-\lambda^{2}\left(x^{2}+y^{2}\right))}.$$

In Equation (15), α, β, and λ are constants for tuning the temporal distribution.

Subsequently, the signal flows containing velocity information from the E-pathways and I-pathways interact within the DPC layer. This process can be described as a linear summation:

(16) S(x,y,t)=E(x,y,t)−a·I(x,y,t)

As synaptic stimuli are not suppressed to give a negative value, a rectified linear unit (ReLU) is introduced to S(x,y,t):

(17) S(x,y,t)=ReLU(S(x,y,t))

where ReLU(x)=max(0,x).

The above modeling of the DPC layer can extract the moving edges of preferred velocity through the interaction between excitation and inhibition.

As shown in Figure 4, the formulation of the DPC layer in the attention process can be defined similarly.

The formulation of E-pathways in the attention process is

(18) $$E_{A}\left(x,y,t\right)=\;\int \int \;P_{AC}\left(u,v,t\right)G_{\sigma_{E}^{\prime }}\left(x-u,y-v\right)dudv$$

The attention kernel is located at the E-pathways to strengthen the excitation of the inter-neurons to the looming power line features and inhibit false line features in the background through the velocity discrimination capacity of the DPC layer. In [25], we have compared three ways of placing the attention kernel in different locations and proved that it achieves the best performance at the E-pathway. Then, the process is

(19) $$E_{A}^{\prime }\left(x,y,t,\theta \right)=\;\int \int \;E_{A}\left(u,v,t\right)W_{A}\left(x-u,y-v,\sigma ,\theta ,\xi \right)dudv$$

The equation of the I-pathways in the attention process is

(20) $$I_{A}\left(x,y,t\right)=\;\int \int \int \;P_{RA}\left(u,v,s\right)W_{\sigma_{I}^{\prime }}\left(x-u,y-v,t-s\right)dudvds$$

where

(21) $$W_{\sigma_{I}^{\prime }}\left(x,y,t\right)=G_{\sigma_{I}^{\prime }}\left(x,y\right)\delta \left(t-\tau \left(x,y\right)\right)$$

The signals from the E-pathways and I-pathways in the attention process similarly interact through a linear summation, and a ReLU then suppresses them:

(22) $$\begin{array}{c} S_{A}\left(x,y,t,\theta \right)=E_{A}^{\prime }\left(x,y,t,\theta \right)-a_{A}\cdot I_{A}\left(x,y,t\right)\\ S_{A}\left(x,y,t,\theta \right)=\mathrm{ReLU}\left(S_{A}\left(x,y,t,\theta \right)\right) \end{array}$$

where θ∈Θ. $S_{A}\left(x,y,t,\theta \right)$ contains the potential line features along the direction θ.

Indeed, the designed attention kernel along θ can not only enhance the regions of lines with identical orientation θ. The linear summation output is further convolved with a directional inhibition kernel $W_{d}\left(\theta \right)$ to inhibit response out of the range $\left(\theta -45^{{}^{\circ}},\theta +45^{{}^{\circ}}\right)$, which is defined as

(23) $$W_{d}\left(\theta \right)=G_{\sigma_{1}}^{\prime }\left(\theta \right)-G_{\sigma_{2}}^{\prime }\left(\theta \right)$$

where $G_{\sigma }^{\prime }\left(\theta \right)$ is the one-dimensional Gaussian function.

The output of the DPC layer after the direction inhibition can be described as

(24) $$S_{A}^{\prime }\left(x,y,t,\theta \right)=\int S_{A}\left(x,y,t,\phi \right)W_{d}\left(\theta -\phi \right)d\phi$$

$S_{A}^{\prime }\left(x,y,t,\theta \right)$ contains the potential line features within the orientation range $(\theta -45^{{}^{\circ}}$, $\theta +45^{{}^{\circ}}$). To detect power lines of any direction in images, we sum $S_{A}^{\prime }\left(x,y,t,\theta \right)$ along the θ-axis direction in Θ. The process can be described as

(25) $$\begin{array}{c} A\left(x,y,t\right)=\sum_{\theta \in \Theta }S_{A}^{\prime }\left(x,y,t,\theta \right) \end{array}$$

where A(x,y,t) is the attention map in Equation (7). Then, the attention map is fed back to the smoothed image R(x,y,t) to enhance the weight of power lines and improve the stimulus response precision [26,33].

#### 2.5.3. G Layer

The grouping layer (G layer) receives the stimuli from the DPC layer. Its functions include enhancing looming-object information, reducing noise in the background, and smoothing the output.

An anisotropic kernel is introduced to improve the line-shape features further. Since the gradients of the power line are not uniform, the anisotropic Gaussian kernel [37] consists of two orthogonal directions and can be described as

(26) $$f\left(x,y,\sigma_{x},\sigma_{y}\right)=k_{2}\cdot \exp\left(-\frac{1}{2}\left(\frac{x^{2}}{\sigma_{x}^{2}}+\frac{y^{2}}{\sigma_{y}^{2}}\right)\right)$$

where $k_{2}$ is a constant coefficient. $\sigma_{x}$ and $\sigma_{y}$ are the standard differences of the *x*-axis and *y*-axis, respectively. The anisotropic Gaussian kernel can also smooth the output by eliminating isolated noise in the background, as it is a deformed Gaussian kernel. Then, the process can be expressed as

(27) $$S_{E}\left(x,y,t\right)=\;\int \int \;S\left(u,v,t\right)f\left(x-u,y-v\right)dudv$$

where $S_{E}\left(x,y,t\right)$ is the line-enhanced information flow. It is then correlated with S(x,y,t) in the form of multiplication to provide a strong response to the looming threats, including power lines, and the grouping correlation result is

(28) $$G\left(x,y,t\right)=S_{E}\left(x,y,t\right)\cdot S\left(x,y,t\right)$$

where G(x,y,t) represents the excitation corresponding to each dendritic cell at the location (x,y) before converging to the axon terminal of the LGMD neuron. The excitation signal of dendrites is followed by a threshold to filter out decayed excitation. That is,

(29) $$\hat{G}\left(x,y,t\right)=\left\{\begin{array}{cc} G(x,y,t) & \mathrm{if}\;G\left(x,y,t\right)⩾T_{de}\left(t\right)\\ 0 & \mathrm{otherwise} \end{array}\right.$$

$T_{de}\left(t\right)$ is the decayed excitation threshold to inactivate the unexcited dendritic cells. $T_{de}\left(t\right)$ is mediated by the side pathway feedforward inhibition (FFI) and is given by

(30) $$T_{de}\left(t\right)=\frac{FFI(t)}{n_{cell}\cdot m}\cdot G_{0}$$

where $G_{0}$ is the baseline threshold, $n_{cell}$ stands for the number of pixels in the input image L(x,y,t), and *m* is a constant. FFI(t) is acquired through the image motion at time $t-T_{0}$, and expressed as

(31) $$FFI\left(t\right)=\;\int \int \;P_{C}\left(x,y,t-T_{0}\right)dxdy$$

Finally, the membrane potential (MP) of the LGMD cell is the summation of all dendritic cells at the terminal. That is,

(32) $$\mathrm{MP}=\;\int \int \;|\hat{G}\left(x,y,t\right)|dxdy$$

The membrane potential can indicate looming threats like power lines and normal-sized objects. It can also trigger dodging behaviors by being input to downstream neurons or the control system of UAVs.

## 3. Experimental Results and Analysis

In this section, we conduct experiments to evaluate the performance of the proposed model in five aspects. Firstly, we analyze location-induced uneven sensibility experimentally and verify the effectiveness of the correction function in addressing this problem in Section 3.2. Secondly, we validate the effectiveness of the attention module in power line detection in Section 3.3. Thirdly, we estimate looming threats for both power lines and normal-sized objects in Section 3.4. Fourthly, we test the detection performance of the model in different contexts in Section 3.5. Fifthly, we evaluate the performance of power line detection comparatively in Section 3.6. Finally, we embed the model into the onboard computer for real-time UAV obstacle avoidance testing in Section 3.7.

### 3.1. Experimental Setup

#### 3.1.1. Dataset Overview

The experiments are conducted using both real-world and synthetic datasets. The synthetic image sequences are generated on the Unreal Engine platform by AirSim [34], which can simulate a drone’s actual flights and produce corresponding video datasets. The real-world datasets are collected from the first-person view (FPV) of a UAV’s actual flights, and both the real-world and synthetic datasets contain FPV data of a drone approaching or colliding with obstacles, including power lines.

#### 3.1.2. Evaluation Metrics

To evaluate the performance of test models in power line detection, we introduced two evaluation metrics: mean intersection over union (MIoU) and processing speed (PS). The two metrics can be expressed as follows:

(33) $$MIoU=\frac{1}{1+K}\sum_{i=0}^{K}\frac{T_{P_{i}}}{T_{P_{i}}+F_{P_{i}}+F_{N_{i}}}$$

(34) $$PS=\frac{\mathrm{NumberOfFrames}}{\mathrm{RunningTime}}$$

where $T_{P}$, $T_{N}$, $F_{P}$, $F_{N}$, and *K* denote the true positive, true negative, false positive, false negative, and the total number of classes, respectively. The MIoU can measure the ability to detect power lines correctly and remove noise simultaneously for the evaluated models. Since UAVs typically fly at high speeds, real-time processing is crucial. Models that can process data quickly give the UAV more time to respond to impending threats. The PS can reveal the real-time capacity of models and is computed in the form of frames per second (FPS), and a high value of PS means high real-time ability.

#### 3.1.3. Data Collection Details

Since we aim to compare the motion vision-based looming-object detector with deep learning-based networks, where the former is based on continuous image sequences and mainly focuses on image motion and the latter on extracting image features in a single image, our dataset comprises constant image sequences. It is designed to contain plenty of looming power lines and apparent image features to fit both. In other words, the looming-object sensitive models only respond to threatening objects, which means the LGMD-based model cannot respond to an object at a distance as it is not considered a threat. In contrast, deep learning networks can only detect target objects with apparent image features. To fairly compare their performance, two strategies are involved to ensure the training for deep learning is sufficient before the test. Both methods are effectively activated in the test set.

Firstly, to ensure the looming-object sensitive models are activated in the test, each frame of the test set contains looming power lines selected from the last 30% of a continuous image sequence observed by a drone flying towards power lines. The first 70% of the images are put into the training set. The division percentage (70% and 30%) is a convention in deep learning. Secondly, we noticed that when drones fly at high speed, it causes the power lines (small-size objects) in images to blur at a distance but be visually apparent only at a relatively close range. Therefore, the explicit pictures and corresponding annotations obtained from the last 30% of the image sequences should be included in the test set as their labels are more accurate for evaluation. Therefore, to compensate for the lack of training data caused by image blur, distinct power line images, and their corresponding labels, are additively included in the training set from other datasets (500 images for training (150 images from other datasets), 150 images for testing). All the annotations of images are finished using LabelMe [38] and V7 Darwin [39].

#### 3.1.4. Parameter Settings

All parameters of the proposed model are tuned empirically. The parameters for all experiment image sequences are listed in Table 1 and remain unchanged. The performance of the proposed SILD and the original D-LGMD model are all tested using the MATLAB software (version R2022b, developed by MathWorks, Natick, MA, USA) on a desktop computer equipped with an Intel-i7 2.9 GHz CPU and 16 GB RAM. Meanwhile, the D-LinkNet model and Hough transform method experiments are conducted using the Microsoft VS Code software (version x64.1.29.0, developed by Microsoft Corporation, Redmond, WA, USA) on the same computer with an NVIDIA 1660 Super GPU and the PyTorch platform.

The quadcopter configuration for the flight experiments in Section 3.7 includes a 450 mm frame wheelbase, with dimensions of 370 mm × 370 mm × 328 mm. It is equipped with a Nora+ autopilot flight control module and an NVIDIA Jetson Orin NX onboard computer. The perception module utilizes a monocular camera, the SIYI A8 mini(manufactured by SIYI Technology, Shenzhen, Guangdong, China). The practical flight experiment of this research uses the Robot Operating System (ROS) for communication between different modules.

### 3.2. Effectiveness of the Location Correction

Early in this research, we found that off-center ambiguous objects easily attract the D-LGMD model, which could affect power line detection. While we have theoretically analyzed that the difference in the location of looming objects can cause an uneven variance in velocity in Section 2.1, the actual situation is more complex [40] than the one described in Figure 3. Therefore, a series of experiments are conducted to first visualize the impact of the location-induced uneven sensibility in a simulated UAV scenario and then evaluate the effectiveness of the proposed location correction method.

**Figure 6:**
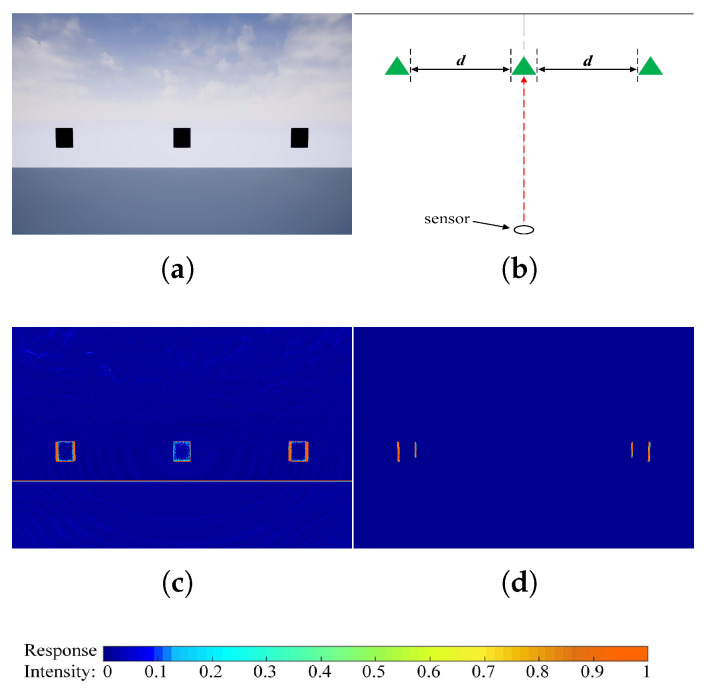
Experiment to verify location-induced uneven sensibility. (**a**) An experimental scene built on AirSim. (**b**) Two-dimensional illustrational description of the experimental scene settings in (**a**). (**c**) P-layer output heatmap of the original D-LGMD model. (**d**) Original D-LGMD model output $\hat{G}\left(x,y,t\right)$ heatmap.

Figure 6 visually demonstrates the impact of location-induced uneven sensitivity. In Figure 6a, a UAV is shown moving toward three identical black squares aligned horizontally. Figure 6b provides a top-down view of the scenario, where the UAV (sensor) follows the trajectory indicated by the red dotted arrow. The central square lies at the center of the sensor’s visual field, while the other two are positioned near the edges at an equal off-center distance, dd. Figure 6c illustrates the initial effect of uneven intensity captured by the photoreceptor (image motion recorded in the P layer). Notably, Figure 6d shows that the D-LGMD visual process exacerbates this disparity: The response to the central square is suppressed due to velocity selection, resulting in an intensified but misleading response to the non-threatening squares.

**Figure 7:**
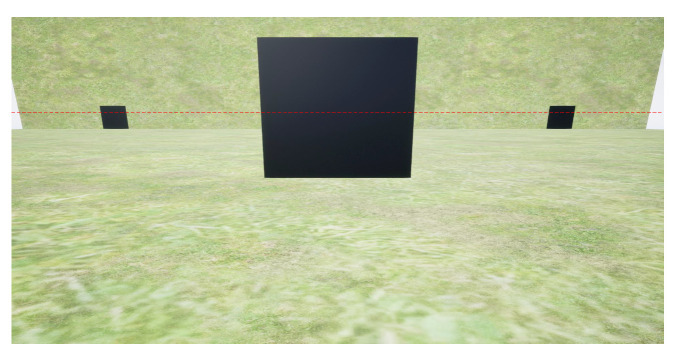
Experiment settings built on AirSim, where a drone flies toward three black square objects of the same size. Note that the square in the center also moves toward the drone to generate strong looming stimuli, while the off-center cubes are static.

**Figure 8:**
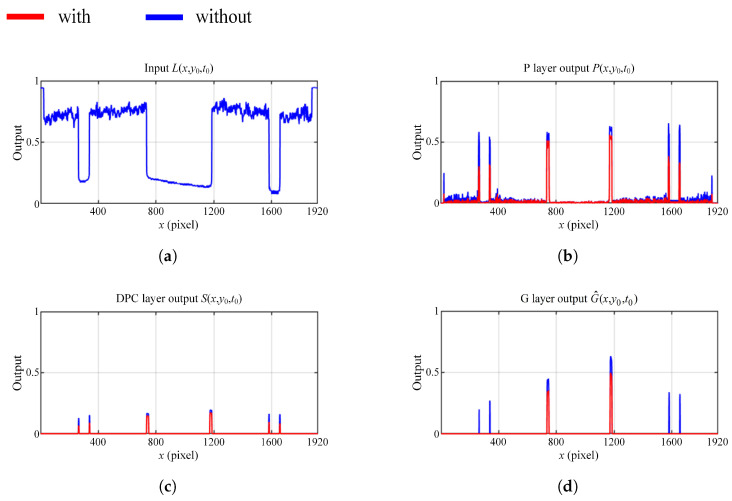
Ablation experiment of location correction. (**a**) Input signal $L(x,y_{0},t_{0})$. Comparative results of (**b**) P-layer output $P(x,y_{0},t_{0})$, (**c**) DPC-layer output $S(x,y_{0},t_{0})$, and (**d**) G-layer output $\hat{G}\left(x,y_{0},t_{0}\right)$ with and without location correction.

**Figure 9:**
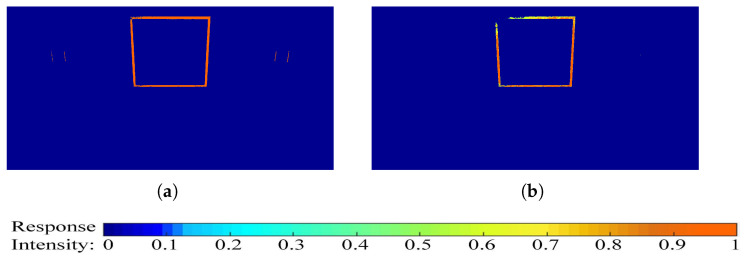
Comparison of G-layer output $\hat{G}\left(x,y,t_{0}\right)$ between the D-LGMD model (**a**) without and (**b**) with the correction function. The intensive response to non-central squares will mislead the D-LGMD to give false alarms without correcting the location.

To test the effectiveness of the location correction in mitigating this impact, an ablation experiment is conducted on D-LGMD (the correction function is universal to reduce the location-induced uneven sensibility and not exclusive to power line detection, so we compared it with the original D-LGMD for general evaluation.) with and without the correction function (Figure 7, Figure 8 and Figure 9). Figure 7 is a sample image (1920×1080) captured by the UAV; in this scenario, the central black square is approaching the UAV on a collision course while the interference squares are located near the boundary of the visual field. Figure 8 presents the model’s pixel-wise response against the location, where pixels are selected along the *x*-axis (denoted by the red dotted line in Figure 7) to focus on the horizontal location. The overall response of the D-LGMD with/without the location correction is visually presented in Figure 9.

Specifically, Figure 8a is the selected input signal $L(x,y_{0},t_{0})$ along the *x*-axis at the horizontal red dotted line ($x,y_{0}=315$) in Figure 7 at the sampled time $t_{0}$. Note the valley in $L(x,y_{0},t_{0})$ denotes the position of the squares. In contrast, Figure 8b–d are responses of the model at each neural layer, and the peaks of intensity indicate the positions of the squares of interest. The P-layer output, shown in Figure 8b, contains motion information with different velocities. Without the correction function, the velocity of the bilateral squares is almost equal to the square in the center. However, after applying the correction function, the velocity of the bilateral squares is largely reduced compared to the central one, resulting in an enlarged velocity difference between threats and non-threats. The DPC-layer output, shown in Figure 8c, filters out low-velocity information but retains high-velocity information, characteristic of the DPC layer. The G-layer output, shown in Figure 8d, can enhance the response to the looming threats received from the DPC-layer output. In contrast, without the correction function the D-LGMD model responds intensively to non-threatening bilateral squares, leading to false alarms despite their small size. The comparative G-layer output is presented in Figure 9, which is consistent with the result in Figure 8d.

### 3.3. Effectiveness of the Line-Attention Module

**Figure 10:**
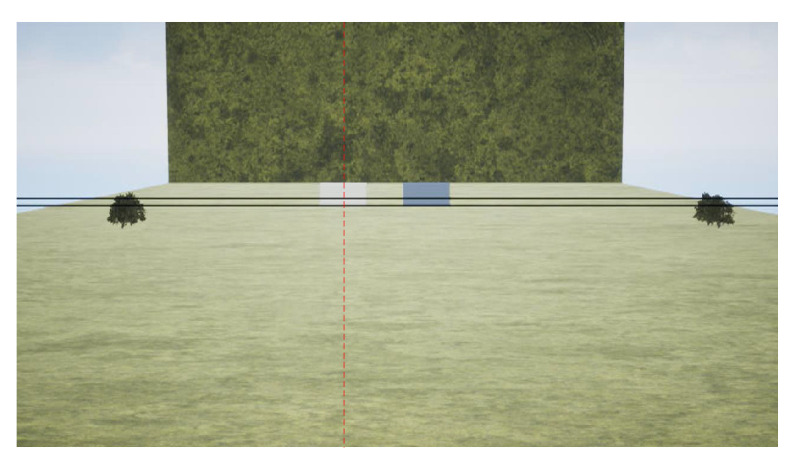
Input image at time $t_{0}$, where there are two power lines at the front and two cubes in the background as interfering noise, and a UAV flies towards the scene.

The primary goal of introducing the attention module is to address the original D-LGMD model’s inability to detect power lines that threaten UAVs. The experiments compare the proposed model with and without the attention module to evaluate the attention module’s effectiveness. The experiment settings and the input image at time $t_{0}$ with a resolution of 1280×720 (width × height) are depicted in Figure 10.

**Figure 11:**
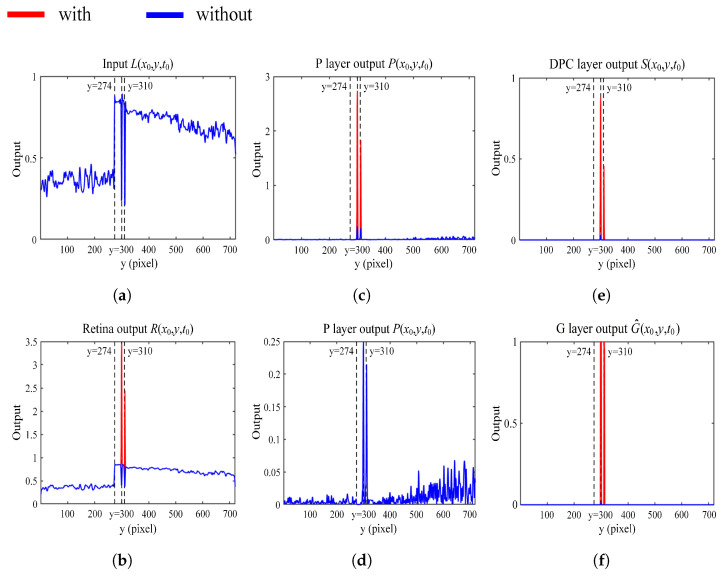
Comparison between the proposed model with and without the attention module. (**a**) Input signal $L(x_{0},y,t_{0})$. (**b**) Comparison of retina output $R(x_{0},y,t_{0})$. (**c**) Comparison of the P-layer output $P(x_{0},y,t_{0})$. (**d**) P-layer output $P(x_{0},y,t_{0})$ of the proposed model without the attention module solely. (**e**) Comparison of the DPC-layer output $S(x_{0},y,t_{0})$. (**f**) Comparison of the G-layer output $\hat{G}\left(x_{0},y,t_{0}\right)$.

To better illustrate how the attention module works in signal processing, we examine the input data and corresponding neural layer outputs along the *y*-axis while fixing *x* at $x_{0}=570$ (indicated by the vertical red dotted line in Figure 10) at a sample time $t=t_{0}$. In Figure 11, we have labeled dotted lines to emphasize three positions along the *y*-axis, namely, *y* = 274, 300, and 310. The location *y* = 274 corresponds to the upper edge of the white cube. We deliberately include edges in the experiment because they are common in reality and are often the most significant sources of noise that affect the performance of power line detection models [41]. The locations *y* = 300 and 310 correspond to the positions of the two power lines, which are the primary targets for detection.

The input signal information, which is the luminance information of the vertical red dotted line shown in Figure 10, is presented in Figure 11a. The luminance changes sharply for the three listed positions (i.e., the upper edge of the white cube and the two power lines) along the *y*-axis. The retina neural output with and without the attention module is shown in Figure 11b; this reflects the intensity of the input image. The attention module selectively and significantly enhances the regions where the power lines are located while ignoring the non-threatening edge (the input pixel intensity value is normalized to the range (0,1); at the same time, the layered output is not limited in this range as the neural process will enhance information of interest pixel-wise).

In the next layer, the information on luminance change is captured by photoreceptors (P layer). Figure 11c compares the resulting photoreceptor neural layer output with/without the attention module. Different from the captured image luminance in Figure 11a, the intensity of looming power lines in the P layer is distinctive because the looming target tends to generate more intensive image velocity in motion vision. After the attention process, the velocity value of the power lines (y=300, y=310) is further increased compared to the response without the attention module. The response to the upper edge of the cube (y=274) is not enhanced since its velocity is too small and it is filtered out in the attention process. Figure 11d is a zoomed presentation of the result without attention enhancement, which helps clarify the response intensity relationship of the three locations. Figure 11e shows the DPC-layer output, which filters out the low-velocity information through the interaction between excitation and inhibition.

### 3.4. Characteristics of the Size-Invariant Looming Detector

**Figure 12:**
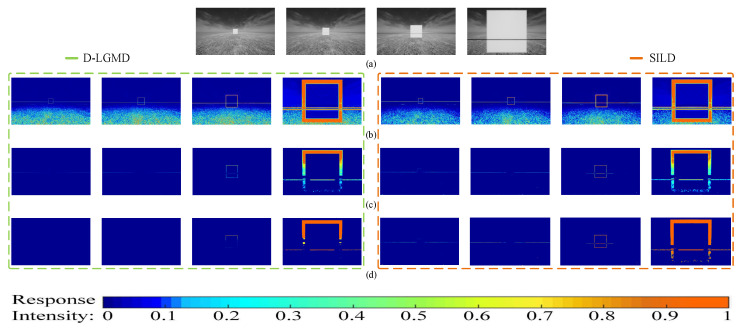
Neural response comparison between the D-LGMD model (left green box) and the proposed SILD (right orange box) in the experiment. (**a**) Grayscale input samples. The comparative output samples of the (**b**) P layer, (**c**) DPC layer, and (**d**) G layer. The whole process lasts 68 frames in the experiment, and the sampled images are at frames 31, 44, 56, and 68.

The attention mechanism in the proposed model is implemented in the additive form, which has been found to outperform the multiplicative one slightly [42]. Moreover, the additive attention mechanism preserves the property of the original model, which guarantees the proposed model can detect both looming power lines and normal-size objects. This size invariant characteristic is tested in a comparative experiment with the original D-LGMD model. As shown in Figure 12, SILD can simultaneously sense the looming power line and the square object. At the same time, the original D-LGMD can only react to the cube when the UAV has almost reached the power line. The neural output images of the P layer, DPC layer, and G layer are transformed into heat maps for intuitive visualization. As seen from Figure 12a, the power line is not visually apparent compared to the white square in the distance due to its narrow width, which illustrates the difficulty in power line detection. Figure 12b shows the P-layer output, which records visual motion in the field of view. It can be observed that the experiment set is complicated since there is interfering noise on the ground, and the P layer allows information on different intensities (i.e., different velocities) to pass. Additionally, line features in our proposed model’s P-layer output are more intensive than in D-LGMD, as the attention module enhances them. Comparing Figure 12b,c, the DPC layer filters out the weak-intensity noise in the background since the DPC layer functions to remove low-velocity (low-intensity) information within the P layer. Although the DPC layer filters out the low-velocity interfering noise, it inevitably weakens the information on looming threats (i.e., the power line and the edges of the white square). As seen in Figure 12d, the G layer strengthens the response intensity of looming threats from the DPC layer in Figure 12c, regathering the focus on looming threats. In addition, from the G-layer output, the D-LGMD model can sense the normal-size looming square, but it cannot detect the power line at the same position until at a reasonably close range (i.e., the last few frames). For instance, in frame 56 of the G-layer response, the D-LGMD model can sense the square but cannot detect the power line at the exact location. This result suggests that the original D-LGMD model cannot detect the power line until it is at a hazardous distance.

In contrast, the proposed model detects the power line as early as frame 31 and robustly at frame 44, whereas the D-LGMD model fails to sense the power line entirely. Notably, the edges of the square are also enhanced by the attention module, as shown in the expanding edges of the square in the P layer (image difference) in Figure 12b at frames 44 and 56. These edges exhibit line-shaped features with velocities close to or exceeding the angular velocity threshold. While the attention mechanism amplifies the edges of the square, it aids the proposed model in detecting looming objects earlier. This is because edges represent object boundaries or changes in surface orientation, which are fundamental properties of objects [43].

**Figure 13:**
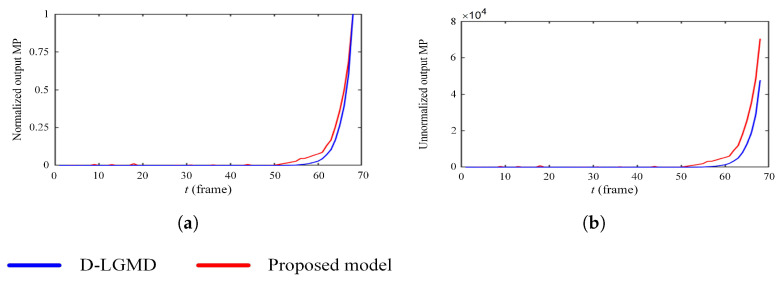
Comparative results of (**a**) normalized output MP and (**b**) unnormalized output MP between the proposed model and the original D-LGMD model.

To quantitatively compare the performance of the proposed model with the D-LGMD model, we analyzed the membrane potential (summation in the G layer) over time (frame), as shown in Figure 13. Figure 13a illustrates the normalized membrane potential (MP) output with respect to time, tt, demonstrating that the proposed model detects imminent collisions earlier than the D-LGMD model. This advantage arises because the proposed model can simultaneously detect both the power line and the square, whereas the D-LGMD model exhibits greater sensitivity to the square than to the power line. Normalization was performed by scaling all MP sequences to the range (0, 1).

In real flight scenarios, the signal controlling the drone’s collision detection system is transmitted in real time, and a warning is triggered when the unnormalized MP surpasses a predefined threshold. Figure 13b compares the unnormalized MP outputs of both models, showing that the proposed model detects the threatening power line more intensively and earlier than the D-LGMD model. Consequently, when an identical threshold is applied to both models during UAV flights, the proposed model provides earlier detection of impending threats, affording UAVs additional time to respond to looming collisions effectively.

### 3.5. Model Performance in Different Contexts

**Figure 14:**
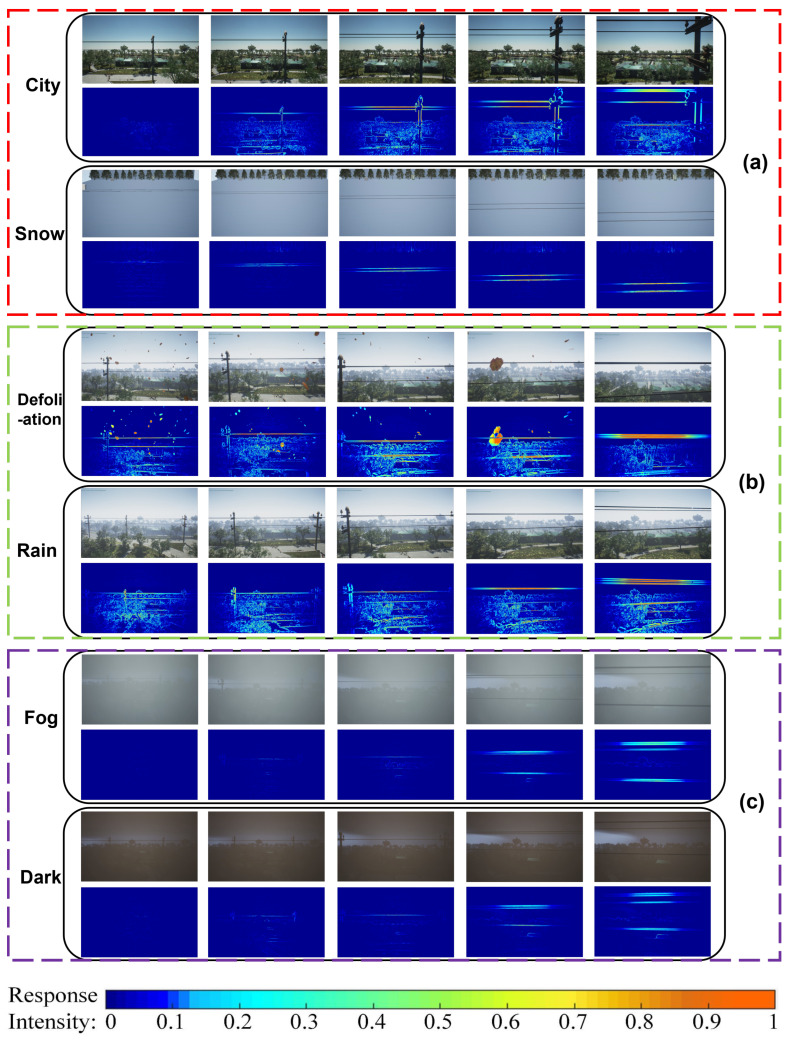
Input images of three comparison groups under different conditions (**top**) with the thermogram output from the proposed model (**bottom**). Group (**a**) shows two background disturbances of city and snow; group (**b**) shows two image noise disturbances of falling leaves and rain; and group (**c**) shows two low-image-texture disturbances of foggy and dark.

A variety of typical and challenging environments need to be selected to evaluate the algorithm’s performance. The selected environments should cover different scenarios and climatic conditions on the Unreal Engine platform by AirSim [34]. These background disturbances have distinct characteristics in terms of complexity. For example, snow, plain (the G-layer output of this scene is covered in Section 3.6, so it is not redisplayed in Figure 14), and cities, which are different colored environments with accompanying environments that may appear as woods, buildings, etc.; these may cause interference. Rainy days and falling leaves may increase the noise in the image, leading to higher false detection rates. Foggy days and low-illumination environments may reduce the features of obstacle texture.

**Figure 15:**
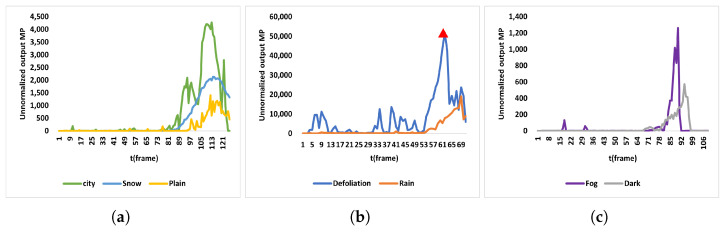
MP output variation curves for the three comparison groups in Figure 14. Figure (**a**) shows the MP output variation under three background disturbances: city, snow, and plain. Figure (**b**) illustrates the MP output variation under two image noise disturbances: falling leaves and rain. Figure (**c**) presents the MP output variation under two low-image-texture disturbances: foggy and dark. Among the three groups, the dashed line shows when the UAV starts to gradually decelerate at that frame, and the red triangle shows the error signal generated by the model being affected by falling leaves.

We analyzed the effect of the background on the model, as shown in Figure 14 and Figure 15. This analysis involves modifying the MP value in the G-layer output to enhance the detection of occluded wires. Specifically, the city dataset includes backgrounds with features at various scales, such as green belts, buildings, and roads, which closely resemble real-world power line scenarios. Additionally, we introduced looming objects (e.g., poles) into the city dataset with scales differing from those of power lines. These elements provide a robust foundation for transitioning the proposed model from simulations to real-world applications.

In Figure 14a, we designed three test scenarios involving looming power lines against different backgrounds: city, snow, and plain. The MP change curves during the looming process, shown in Figure 15a, demonstrate that in all three backgrounds the UAV exhibits a clear membrane potential output as it approaches the power line. Notably, the black power line against the snowy background produces a smoother MP output curve due to the high contrast between the power line and the background. Conversely, the complexity of the urban scenario is reflected in its MP change curve, which is less smooth due to the diverse features present in the scene. Furthermore, the thermogram of the G-layer output in the urban setting illustrates the model’s ability to detect looming objects of varying scales simultaneously.

In Figure 14b, we introduced two types of image noise—falling leaves and rain—superimposed on the urban scene. The MP change curves during the looming process, shown in Figure 15b, indicate that despite these noise disturbances, the UAV can still detect the power line as it approaches a collision. However, the average MP output value is notably higher under these conditions. Specifically, more random interference, such as falling leaves, significantly impacts the model’s ability to detect looming dangerous objects, reflecting the challenge posed by dynamic and unpredictable noise.

In Figure 14c, we introduced two image disturbances—foggy and dark conditions—over the urban scene, which diminish the texture features of the looming object in the image. The MP change curve during the looming process, shown in Figure 15c, reveals that as the UAV approaches the power line, a distinct peak remains visible on the curve. However, the peak is higher than in cases without image interference and normal illumination. This increase reflects the impact of reduced texture features, as the MP output remains close to zero except for the moments when the UAV is about to collide with the power line.

### 3.6. Comparison of the Real-World and Synthetic Datasets

**Figure 16:**
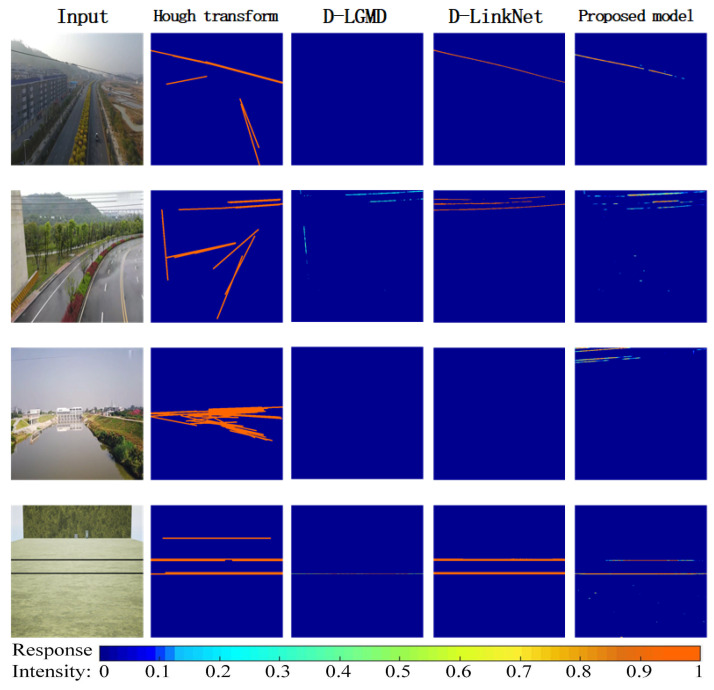
Example results of the four models. The inputs of the first three lines are from real-world datasets, and the input of the last line is generated on the AirSim [34] platform.

Since power line detection is one of the most challenging and essential tasks in UAV-based power line detection [44], we have conducted a comparative experiment between our proposed model, the D-LGMD model [24], D-LinkNet [8], and the Hough transform [45] in power line detection on the real-world and synthetic datasets. The input image size in the experiment was 512×512 pixels, and the comparative experiment evaluated the performance of the four models, as shown in Figure 16 and quantitatively evaluated in Table 2. The input images of the D-LGMD model, Hough transform, and our proposed model are single-channel, while the input images of the D-LinkNet are three-channel (RGB). As seen in Figure 16, D-LinkNet achieved the best performance in accurately detecting power lines, while our proposed model was slightly inferior to D-LinkNet (the MIoU of the proposed model was only 4.97% lower than that of D-LinkNet, but higher than that of the Hough transform by 40.7% and D-LGMD by 34.6%). In contrast, the Hough transform was easily confused by noise lines in the background, and D-LGMD sometimes sensed the looming power lines weakly. Note that the Hough transform and D-LinkNet outputs were binarized without the sensibility to collision threats. In contrast, the output intensity of the D-LGMD and our proposed model was directly associated with the threat level of looming objects. The metric MIoU, representing the detection accuracy for power lines, showed that our model was comparable to the state-of-the-art deep learning model D-LinkNet and superior to both the Hough transform method and the D-LGMD model, which is consistent with the results visualized in Figure 16. The proposed model, the D-LGMD model, and the Hough transform method greatly exceed D-LinkNet in processing time, as shown in the metric PS, computed by averaging over ten times over 100 images and expressed as FPS. (The proposed model’s number of FPS is 66.6% lower than that of the Hough transform, 45.4% lower than that of D-LGMD, and 28.905 times lower than that of D-LinkNet). Combining the two metrics, we conclude that our proposed model can simultaneously detect power lines correctly, process images in a timely fashion, and sense looming threats without bias based on size, making it perfectly applicable for drones’ safe flight in power line detection scenarios.

### 3.7. Real-World Flight Validation

**Figure 17:**
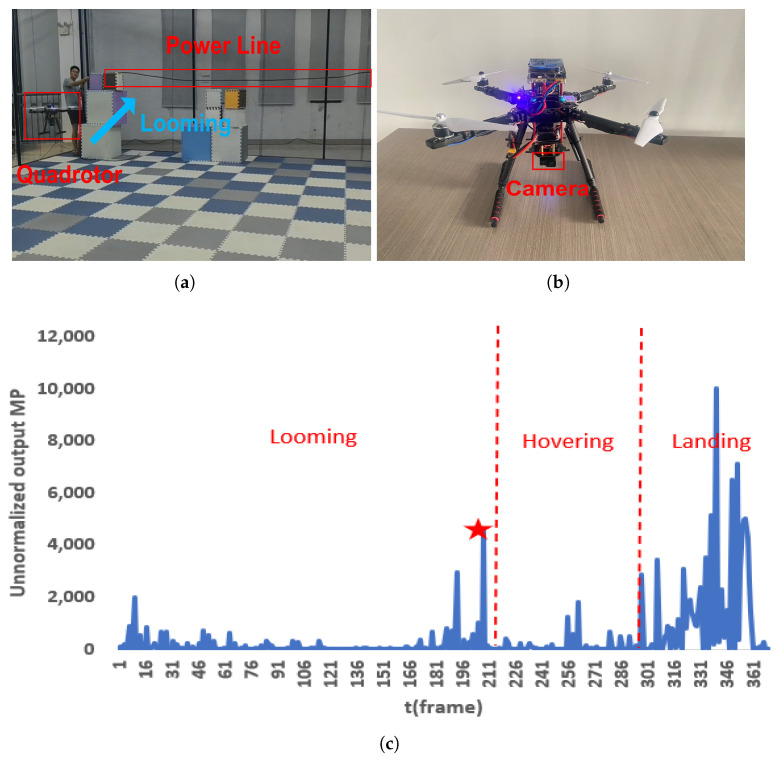
Experimental results of the proposed system. (**a**) Experimental site; (**b**) quadrotor obstacle avoidance system for power line; (**c**) MP output variation curves during actual flight. The red pentagram shows when the MP value exceeds the threshold (set to 4000), triggering the drone to autonomously hover and land.

We deployed the proposed obstacle avoidance model on an embedded computing platform, NVIDIA Orin NX, and integrated it with a monocular camera pod, G1, to construct a quadrotor obstacle avoidance system, as shown in Figure 17b. At the experimental site, depicted in Figure 17a, the quadrotor was operated under off-board control. During the flight, when the system detected a looming power line ahead, it triggered the off-board control to execute an emergency hover-and-landing operation based on the membrane potential (MP > 4000) output from the model. If no obstacle was detected, the quadrotor performed a return operation, guided by the starting-point coordinates provided by the motion capture system. The experimental results demonstrate that the proposed model effectively addresses the obstacle avoidance problem in simple power line scenarios.

To illustrate the process and results of this experiment, a demonstration video showcasing our approach is available at https://www.youtube.com/watch?v=JhRmxwUzkiE (accessed on 25 December 2024). The video includes a third-person perspective of the experiment, the UAV’s first-person view (i.e., the model’s input), and the model’s output. This visualization is intended to help readers better understand the proposed method and its performance.

During the quadcopter power line detection process, the model successfully identified power lines above the detection threshold. A background black column approximately 0.5 m away from the power line was also detected, albeit with a lower MP than the power line, indicating potential parameter optimization issues. Despite this, the experiment validated the model’s capability to capture cross-size attention effectively.

## 4. Discussion

### 4.1. Impact of Location Correction and Line-Attention Module

This phenomenon seems to exist in human beings, and some researchers have found that humans are more sensitive to stimuli looming from the periphery than from the center [46]. However, this problem has not been thoroughly investigated in LGMD modeling. In Section 3.2, the results of the P-layer output indicate the correction function cannot eliminate the uneven input intensity caused by looming-object location. Still, it is compatible with the signal process of the D-LGMD model. As we can see, the centered threat only activates the final spatial response after being further filtered by the DPC and G layers. Overall, the proposed location correction can effectively gather the attention of D-LGMD to center threats, which is essential when navigating complex environments with multiple near-miss objects.

As can be seen from Section 3.3, without the attention module, it is difficult for the DPC layer to react to the looming power lines. Finally, Figure 11f gives the output of the G layer, which is further enhanced with hidden information compared to the output of the DPC layer. Therefore, the model with the attention mechanism can perceive the power lines more centrally compared to the model without the attention mechanism.

### 4.2. Analysis of the Size-Invariant Looming Detector

Attention mechanisms are commonly seen in animals, and they can help focus limited computation resources on salient regions or interested targets amid alternative distractions in a world abundant with information [47]. It can help animals forage, avoid predators, and seek mates [48]. For instance, mantises can selectively track one prey among many [49], flies can direct their behavior towards stimuli in specific parts of their visual field [50], and female crickets can adjust to the frequency of male mating calls [51].

In small-target detection, the attention mechanism plays a critical role [52,53,54]. Wang [54] proposed a prediction mechanism and feedback loop to facilitate the attention module, achieving excellent performance in detecting small moving targets in complex scenarios. Detecting multi-scale objects ranging from extremely small to average size simultaneously in one neural network is still challenging for robotics and computer vision [55,56,57]. Drawing on the above neural mechanisms, we propose a model that uses additive attention in a feedback loop and achieves size-invariant looming sensitivity for power line detection scenarios.

We discuss the key characteristics of the proposed model and its performance in different aspects. First, the additive attention mechanism in the model provides a subtle advantage over the traditional multiplicative form, particularly in its ability to enhance the perception of weak signals while maintaining the original features of the model. By assigning different weights to input features, the additive attention mechanism amplifies responses to weak signals without compromising the detection of normal objects, which is crucial for multi-target detection in complex environments. Second, the model demonstrates robust size invariance, enabling it to detect both faintly visible power lines and normal-sized objects within the same environment. With the attention mechanism, the model not only detects distant power lines but also identifies potential threats more efficiently. Additionally, the additive attention mechanism enhances the edge features of power lines, which allows the model to better distinguish between different objects and improve detection accuracy. Finally, early detection of looming threats is critical for collision avoidance. The introduced additive attention mechanism enables the model to detect and respond to potential threats more promptly and accurately in complex environments, contributing to the safety of UAVs.

### 4.3. Bionic Vision-Based Autonomous Control of UAVs in Power Line Scenarios

In Section 3.5, we observe that, despite interference from varying scenes, image noise, and low-texture features, the model is still capable of detecting looming objects of different scales, such as power lines and poles, to a certain extent. Although the peak mean precision (MP) values differ across experimental conditions, the results provide valuable insights for applying the model in UAV obstacle avoidance within power scenarios. In Section 3.6, we present experimental evaluations on both real-world and synthetic datasets. Power line detection is a critical and challenging task for UAVs, especially in complex environments, where power lines are often difficult to distinguish from background noise. Therefore, selecting an appropriate model for efficient and accurate power line detection is essential to ensure safe UAV operations. The proposed model performs well in power line detection, not only achieving comparable accuracy to the advanced deep learning model D-LinkNet but also surpassing it in certain aspects. Additionally, the model effectively senses looming threats and processes image data in real time, mitigating bias introduced by variations in object size or background noise. Considering the trade-off between detection accuracy and real-time processing capabilities, our model is well suited for UAV-based power line detection in complex environments, ensuring the safe flight of UAVs.

However, in the real-world flight experiments conducted in Section 3.7, we observed that the trigger conditions and off-board control mechanisms still require further refinement. The current trigger condition is designed based on the experimental results in Section 3.5, specifically relying on the fact that a higher MP output is generated when the power line is sufficiently close to the UAV. While this mechanism performs well in some experiments, repeated trials revealed several issues with the threshold setting. If the threshold is too low, the system triggers off-board control when the UAV is still far from the power line, resulting in unnecessary interventions. Conversely, if the threshold is set too high, the system may fail to trigger hovering in time, causing the UAV to enter return mode prematurely.

As shown in Figure 14, the proposed algorithm demonstrates excellent performance on the simulated dataset (AirSim data). However, as indicated in Figure 16, its performance on real-world datasets and during actual flight tests is significantly lower, despite multiple parameter adjustments. This discrepancy can be attributed to several factors. Firstly, the brightness levels in the simulated and real-world datasets differ substantially. Specifically, the indoor experimental environment during flight tests exhibited illumination levels ranging from 14.4 lux to 477 lux, in contrast to outdoor conditions, where illumination can exceed 100,000 lux. Although binary processing was applied at the P and G layers to mitigate the effects of brightness variation, the improvement was limited, highlighting the need for further research into brightness adaptation. Subsequent studies consider methods for remapping the input image using image moments [23]. Secondly, the real-world dataset presents more complex background interference compared to the simulated dataset, further affecting algorithm performance. Additionally, due to limitations in drone flight control technology, unpredictable self-induced oscillations may occur during autonomous flights, a phenomenon absent in simulations, posing additional challenges to algorithm stability. Therefore, while the algorithm performs well on simulated data, its adaptability to real-world scenarios requires further optimization, which will be a focus of future work.

The rationality of the off-board control mechanisms also requires further evaluation. In practical applications, a UAV utilizing visual obstacle avoidance may be unable to continue its return mission upon detecting a looming power line, triggering a hover landing. If a strategy of avoiding the power line is employed, the UAV may face the risk of collision with other obstacles (e.g., power lines, electric poles, trees). Therefore, the monocular camera-based obstacle avoidance strategy may have limitations in complex power line environments. Specifically, in real-world power line scenarios, this approach may not be as effective as intended. As discussed in Section 3.3, without the attention module, the DPC layer struggles to react to looming power lines. In contrast, the output from the G layer, as shown in Figure 11f, is enhanced with hidden information, offering improved performance compared to the DPC layer. Thus, the model with the attention mechanism is better able to perceive power lines, providing a more focused detection compared to the model without the attention mechanism. Additionally, we recorded the MP changes in both the image and numerical values of the UAV during hovering and landing in each experiment, offering valuable insights for further investigation into UAV control strategies under bio-inspired vision.

Finally, we raise some concerns regarding the necessity of the G layer in the proposed model. In the LGMD model [20], the G layer is designed to filter background noise and enhance the features of looming objects. Removing the G layer sacrifices some of this enhancement, leading to the question of whether a UAV with high maneuverability can better understand the relationship between the environment and looming objects, and whether this could aid in autonomous obstacle avoidance. This aspect requires further investigation. In our experiments, removing the G layer resulted in an increase in background object contours (non-looming objects) and noise in the output. Future work will explore the integration of motion segmentation techniques [58] following the removal of the G layer, with the goal of providing more accurate obstacle avoidance strategies for UAVs based on bio-inspired vision.

## 5. Conclusions

This paper presents a visual system for complex UAV-based power line detection scenarios. Leveraging a bio-inspired looming-object detector along with an attention and feedback structure, our model addresses the challenging task of detecting multi-scale objects, ranging from extremely small to normal sizes. To mitigate interference from near-miss objects during detection, we propose a position correction mechanism that enhances detection accuracy. To systematically evaluate our approach against other motion vision methods, including deep learning-based techniques, we designed a small dataset consisting of continuous FPV image sequences for the power line detection task. Additionally, we assessed the performance of the proposed model under various conditions, including different backgrounds, image noise, and low-texture features. The model was also ported to a UAV-embedded board, successfully performing a basic collision avoidance task in a power line scenario. The experimental results demonstrate that the motion vision-based swipe detector excels in pixel-level power line detection, computational efficiency, and collision perception. Our research offers a computationally efficient solution for size-invariant looming-object detection in complex power line environments, which holds significant potential for applications in computer vision and UAV-based power line detection.

The LGMD model demonstrates significant scalability in handling diverse scenarios. Traditionally, LGMD has been considered limited, as it struggles to detect looming objects in certain special conditions, such as when the contour edges are small, the speed is slow, or the contrast is low. In these cases, the model may fail to perceive approaching objects and, when extended to detect such special objects, may generate false positive responses to background motion. To address these challenges, we propose selectively enhancing motion visual features that are characteristic of specific objects, such as the unique shape of power lines. Additionally, feature aggregation is applied to make the enhanced features more coherent and less sparse. These augmented features are then fed back as intermediate inputs to the LGMD, improving its sensitivity and robustness in detecting objects in complex dynamic environments. Our approach reveals that LGMD has such an extensibility that the perception of specific targets can be enhanced by exploiting the shape features of the particular target without violating the basic assumptions of the LGMD model for background motion filtering.

To further improve model performance, future optimizations will focus on the following aspects: First, in the design of trigger conditions, image processing techniques such as feature extraction and motion segmentation based on the G-layer output will be explored to enhance the model’s accuracy in detecting power lines. Second, in the off-board control strategy, measures will be taken to prevent premature landing triggers by increasing flight altitude, allowing the UAV to continue its return mission. Additionally, given the model’s low computational resource requirements, future work will explore expanding obstacle avoidance capabilities by integrating cameras in multiple directions or using panoramic cameras, thus improving the system’s robustness and adaptability. 

## Figures and Tables

**Table 1:** Parameters of the proposed visual system.

Equation	Parameters
(3)	$\sigma_{P}=1$
(5)	A=2,B=3
(6)	$\sigma =2.0,\;\Theta =\left\{0^{{}^{\circ}},\;90^{{}^{\circ}}\right\},\;\xi =0.4$
(10)	$k_{1}=1.0$, n=1.3
(15)	α=−0.1,β=0.5,λ=0.7
(23)	$\sigma_{1}=0.5,\;\sigma_{2}=1.0$
(26)	$k_{2}=1.0,\;\sigma_{x}=6,\;\sigma_{y}=2$
(30)	$m=0.4,\;G_{0}=0.5$
(33)	K=1

**Table 2:** Performance of the tested models.

	*MIoU*	*PS* (FPS)
Hough transform [45]	0.5452	92.5926
D-LGMD [24]	0.5697	56.3552 (For looming-object detection, it has been verified that the image size can be further compacted while not harming the performance of the D-LGMD looming detector [24]. It was achieved on a 480 MHz embedded MCU to perform a 70 FPS rate for looming-object detection with 80 × 60 image size. This indicates the proposed method can also perform a comparable low-power calculation on embedded systems.)
D-LinkNet [8]	0.8070	1.1052
Proposed model	0.7667	30.8587

## Data Availability

The original contributions presented in this study are included in the article material. Further inquiries can be directed to the corresponding author.
